# Impact of Specific Metabolic Syndrome Combinations on Model-Estimated 10-Year Cardiovascular Risk in a Taiwanese Population

**DOI:** 10.3390/jcm15114075

**Published:** 2026-05-25

**Authors:** Tsung-Min Yeh, Kuang-Chen Hung, Chia-Lien Hung, Chih-Li Lin, Shih-Kai Tu, Yu-Tse Tsan, Chun-Cheng Liao

**Affiliations:** 1 Department of Family Medicine, Taichung Armed Forces General Hospital, No. 348, Sec. 2, Zhongshan Road, Taiping District, Taichung City 411, Taiwan; amin6231@gmail.com (T.-M.Y.); k98dddsss@yahoo.com.tw (S.-K.T.); 2 Division of Occupational Medicine, Department of Emergency Medicine, Taichung Veterans General Hospital, No. 1650, Sec. 4, Taiwan Boulevard, Xitun District, Taichung City 407, Taiwan; janyuhjer@gmail.com; 3 Department of Surgery, Kaohsiung Veterans General Hospital, No. 386, Dazhong 1st Road, Zuoying District, Kaohsiung City 813, Taiwan; syou@vghks.gov.tw; 4 College of Nursing, Meiho University, No. 23, Pingguang Road, Neipu Township, Pingtung County 912, Taiwan; 5 College of Health Sciences, Central Taiwan University of Science and Technology, No. 666, Buzih Lane, Beitun District, Taichung City 406, Taiwan; 6 School of Medicine, National Defense Medical University, No. 161, Sec. 6, Minquan East Road, Neihu District, Taipei City 114, Taiwan; 7 Department of Medical Education and Research, Taichung Armed Forces General Hospital, No. 348, Sec. 2, Zhongshan Road, Taiping District, Taichung City 411, Taiwan; chia-lien@803.org.tw; 8 Institute of Medicine, Chung Shan Medical University, No. 110, Sec. 1, Jianguo North Road, South District, Taichung City 402, Taiwan; dll@csmu.edu.tw; 9 School of Medicine, Chung Shan Medical University, Taichung City 402, Taiwan

**Keywords:** metabolic syndrome, MetS component combinations, cardiovascular risk, Framingham risk score, ASCVD

## Abstract

**Background:** Metabolic syndrome (MetS) affects over 30% of the global population and is closely linked to higher cardiovascular (CV) morbidity and mortality. Although MetS is recognized as a significant CV risk factor, limited studies have examined which specific combinations of MetS components are associated with long-term predicted CV risk. Furthermore, limited evidence exists using established 10-year CV risk-prediction models in Asian populations. **Methods:** We analyzed data from 111,695 Taiwanese adults aged 30–75 years who underwent health screenings from 2007 to 2022. Predicted CV risk was estimated using the Framingham Risk Score (FRS) and Atherosclerotic Cardiovascular Disease (ASCVD) Risk Estimator at baseline and at 5- and 10-year follow-ups. Cox regression models adjusted for clinical variables were applied to evaluate the association between different MetS patterns and progression in estimated 10-year CV risk. **Results:** Of the 111,695 participants, 4435 had persistent MetS with the same exact three components at both baseline and follow-up. Among the MetS combinations, the TFB pattern (elevated triglycerides, fasting glucose, and blood pressure) was consistently associated with greater progression in predicted 10-year CV risk over 5- and 10-years follow-up periods in both the FRS (HR = 1.189–1.204) and ASCVD (HR = 1.144–1.146) models (all *p* < 0.05). Although the effect sizes were modest, the associations were consistent across models and time points. **Conclusions:** The TFB pattern was consistently associated with greater progression in predicted 10-year cardiovascular risk across both the FRS and ASCVD models. These findings suggest that evaluating specific MetS patterns may provide additional value beyond the total number of components and may help clinicians prioritize high-risk individuals for targeted screening and early intervention.

## 1. Introduction

Metabolic syndrome (MetS) has a global prevalence estimated between 31.5–34.6% [1], and it exhibits a strong association with cardiovascular (CV) risk. Recent studies have shown that MetS elevates the likelihood of developing coronary artery disease, dying from congestive heart failure, and experiencing all-cause mortality [2,3]. Furthermore, a 13-year prospective study by Guembe et al., involving a Mediterranean population-based cohort (aged 35–84 years), demonstrated that for each additional MetS trait, the incidence of actual major cardiovascular events relatively increased by approximately 22% (HR = 1.22, 95% CI: 1.09–1.36) [4]. Dekker et al. and Mottillo et al. further demonstrated that MetS is linked to a two-fold increase in CV outcomes and a 1.5-fold rise in all-cause mortality [5]. In addition to the classical components of metabolic syndrome, biochemical markers may also contribute to its underlying pathophysiology. Previous studies have shown that the atherogenic index of plasma [log(TG/HDL-C)], waist circumference, systolic blood pressure, glucose, and alanine aminotransferase are associated with MetS manifestation, supporting its multifactorial metabolic nature [6]. Most existing studies focus on the cumulative number of MetS components, assuming that each component contributes equally to cardiovascular risk. However, different combinations of components may reflect distinct pathophysiological mechanisms and result in heterogeneous cardiovascular risk profiles. In addition, traditional risk models typically treat individual risk factors independently and may not fully capture potential interactions or synergistic effects among MetS components.

Cardiovascular disease (CVD) continues to be a major contributor to global morbidity and mortality [7]. Over recent decades, multiple risk prediction models have been developed to estimate an individual’s predicted risk of future CVD events and to support early preventive measures in asymptomatic populations. Since the launch of the Framingham Risk Score (FRS) in 1998, various region- or population-specific tools have emerged, including the revised FRS (2008), the Atherosclerotic Cardiovascular Disease (ASCVD) Risk Estimator, the JBS3 risk calculator (2014), the China-PAR algorithm for ASCVD risk estimation (2016) [8], the QRISK3 score (2017), and the SCORE2-OP calculator (2021). However, the applicability of these risk prediction models to Asian populations may vary, as many were originally developed in Western cohorts and may not be fully calibrated for Taiwanese individuals. This highlights the importance of evaluating these models in Taiwanese populations.

Despite the availability of various models, there is still no consensus on the most suitable risk calculator for the Taiwanese population. In addition, although extensive research has examined the association between MetS and observed CV events, fewer studies have evaluated its relationship with model-estimated cardiovascular risk using established prediction tools such as the FRS and ASCVD Risk Estimator. This research gap underscores the need to evaluate MetS using established CV risk-prediction models in a Taiwanese cohort.

While MetS is widely acknowledged as a major factor contributing to increased CV risk, few studies have examined how specific combinations of MetS components influence long-term predicted CV risk. Most previous studies have relied on cross-sectional or single-time-point designs, which do not capture temporal changes or the cumulative effects of MetS. These approaches may also overlook potential interactions between components. In contrast, our longitudinal design with 5- and 10-year follow-up enables the evaluation of changes in predicted CV risk over time and better characterizes the impact of specific MetS component combinations. Therefore, this study aimed to evaluate the associations between various MetS component patterns and estimated 10-year cardiovascular risk in a Taiwanese population using both baseline and longitudinal analyses. We hypothesized that specific MetS component combinations would be associated with greater progression in predicted cardiovascular risk.

## 2. Materials and Methods

### 2.1. Study Population

This retrospective longitudinal study analyzed data collected between 2007 and 2022 by the MJ Health Screening Center, a large private health examination organization in Taiwan that includes participants from multiple regions and diverse socioeconomic backgrounds; although, it may not be fully representative of the general Taiwanese population. Participants aged 30–75 years at their initial examination were included, consistent with the age range applicable to the FRS calculator.

Exclusion criteria included individuals younger than 30 or older than 75 years, as well as those with incomplete data required for predicted CV risk calculation. Specifically, cases missing key variables—such as age, sex, race, history of diabetes mellitus or hypertension, use of antihypertensive medications, systolic blood pressure, triglycerides, high-density lipoprotein cholesterol (HDL-C), low-density lipoprotein cholesterol (LDL-C), fasting glucose, body mass index (BMI), or smoking status—were excluded from the analysis.

All identifying information was removed by the MJ Health Research Foundation before data release. Written informed consent was obtained from each participant at the time of screening, authorizing the use of de-identified data for research. The study received ethical approval from the Institutional Review Board of Tri-Service General Hospital, Taiwan (IRB No. A202005160) and was conducted in accordance with the Declaration of Helsinki. Data access was formally approved by the Taichung Armed Forces General Hospital and the MJ Health Research Foundation (authorization code, MJHRF2021003A).

Data were drawn from the MJ Health Screening Database from 2007 to 2022 (*n* = 148,290). Participants were excluded if data for either the FRS (2008) or the ASCVD Risk Estimator (2013) were missing (*n* = 20,812) or if they were under 30 or over 75 years old (*n* = 15,783). Participants with inconsistent MetS component combinations between baseline and follow-up were also excluded to focus on persistent metabolic patterns and reduce misclassification, although this approach may introduce selection bias. The final analysis included 57,996 participants. Primary pattern-based analyses focused on participants with exact three-component MetS combinations. For the analyses, participants with four (*n* = 1994) or five (*n* = 605) MetS components were excluded, resulting in an analytic sample of 55,397 participants (Appendix A). Additional analyses were conducted separately for participants with four-component MetS combinations to evaluate whether similar associations were observed in more complex MetS patterns. Participants with five-component MetS were not included in pattern-specific subgroup analyses because of the limited sample size and the absence of component variability.

### 2.2. Clinical and Laboratory Assessment

All participants received standardized physical examinations, anthropometric measurements, and fasting blood tests as part of routine health screenings. Systolic and diastolic blood pressures were recorded by trained personnel using standardized calibrated devices according to the MJ Health Screening Center protocol. BMI was determined by dividing weight in kilograms by height in meters squared (kg/m^2^).

Fasting blood samples were collected to measure triglycerides, glucose, uric acid (UA), HDL-C, and LDL-C, with results reported in mg/dL. Participants also completed a detailed, self-administered questionnaire covering their medical history, current use of antihypertensive or oral hypoglycemic medications, and smoking status. Standardization of data collection across centers was ensured through uniform protocols, trained personnel, identical equipment, and periodic device calibration.

### 2.3. Definition of the MetS

Based on previous studies in Asian populations, the modified NCEP ATP III criteria were selected because they incorporate ethnicity-specific waist circumference thresholds and have been widely used in epidemiological studies of MetS in Asian cohorts. Prior comparisons have also suggested that these criteria may provide better discrimination of cardiometabolic risk than some alternative definitions in certain Asian populations [9,10]. According to this guideline, MetS is diagnosed when at least three of the following five criteria are met:

1. **Central obesity**: Waist circumference ≥ 90 cm for men or ≥80 cm for women.
2. **Hypertriglyceridemia**: Triglyceride levels ≥ 150 mg/dL (1.7 mmol/L), or receiving specific medication to treat this lipid abnormality.
3. **Low HDL-C**: HDL-C < 40 mg/dL (1.03 mmol/L) in men or <50 mg/dL (1.29 mmol/L) in women, or receiving treatment for low HDL-C.
4. **Elevated blood pressure**: Systolic blood pressure ≥ 130 mm Hg and/or diastolic blood pressure ≥ 85 mm Hg, or taking antihypertensive medication.
5. **Impaired fasting glucose**: Fasting plasma glucose ≥ 100 mg/dL (5.6 mmol/L), or using medication for high glucose levels.

MetS component patterns were denoted as combinations of triglycerides (T), fasting glucose (F), waist circumference (W), HDL-C (H), and blood pressure (B).

#### Cardiovascular Risk Assessment and Risk Classification

Predicted cardiovascular risk was estimated using the Framingham Risk Score (FRS) and the 2013 ACC/AHA ASCVD Risk Estimator according to their published original equations and standard model variables [11,12]. Although these models were not specifically developed for Taiwanese populations, they are widely used risk assessment tools that allow standardized cardiovascular risk estimation and comparison across studies [11,12]. Previous studies have suggested that these models may show variable calibration in Asian populations. These two models were chosen for their wide clinical adoption, similar periods of publication, and compatibility with the data available for this analysis. Moreover, all variables required for these risk scores could be easily collected through routine, cost-effective clinical tests.

To support clinical interpretation and risk stratification, participants’ estimated 10-year CV risk percentages were grouped into standard risk categories according to existing guidelines. For the FRS, individuals were classified as low risk (<10%), intermediate risk (10–<20%), or high risk (≥20%) [13]. For the ASCVD Risk Estimator, a more detailed classification was used: low risk (<5%), borderline risk (5–7.5%), intermediate risk (7.5–20%), and high risk (≥20%) [14].

### 2.4. Outcomes

The primary outcome of this study was the change in predicted 10-year CV risk over time. We evaluated whether predicted cardiovascular risk increased at follow-up assessments conducted approximately 5 and 10 years after baseline. Participants were divided into two groups based on the difference between their estimated risks at these two time points. Individuals whose second predicted CV risk was higher than their baseline value (i.e., risk difference > 0%) were placed in the progression group, indicating higher predicted risk at follow-up. Those whose predicted risk remained the same or decreased (i.e., risk difference ≤ 0%) were classified as the non-progression group. Changes in predicted cardiovascular risk were used as a surrogate endpoint, as such estimates are commonly employed for risk stratification and the development of preventive strategies in asymptomatic populations. The dichotomization of risk change was adopted to facilitate clinically meaningful interpretation of risk progression.

### 2.5. Statistical Analysis

Independent *t*-tests were performed to compare changes in predicted CV risk, UA, and LDL-C levels between participants with and without predicted risk progression, as determined by the FRS or ASCVD model. The Pearson chi-squared test was used to compare sex, age group, BMI, and MetS component combinations according to predicted risk progression status and predicted CV risk categories. Linear regression was applied to evaluate associations between individual MetS components or the total number of MetS criteria and changes in estimated CV risk based on the FRS or ASCVD. Cox proportional hazards models were used to estimate the associations between MetS component patterns and progression in predicted 10-year cardiovascular risk over the follow-up intervals. These HRs were adjusted for age, sex, BMI, UA, LDL, baseline predicted CV risk, and the initial risk category according to each model. Statistical analyses were conducted using SPSS 22.0 (IBM, Armonk, NY, USA). A *p*-value of <0.05 was considered statistically significant.

## 3. Results

### 3.1. Participant Characteristics and Baseline MetS Combinations Across Risk Categories

Table 1 presents the baseline characteristics of participants according to predicted risk progression status, as determined by the FRS and the ASCVD Risk Estimator. Participants in the progression group showed significantly greater increases in predicted cardiovascular risk compared with those in the non-progression group. Sex, age groups, BMI, UA, and LDL-C differed significantly between the progression and non-progression groups for both the FRS and ASCVD Risk Estimator (*p* < 0.001).

Table 2 shows the distribution of MetS component combinations across the FRS and ASCVD risk categories. A total of 57,996 participants met the study eligibility criteria. For analyses of three-component MetS patterns shown in Table 2, participants with four or five MetS components were excluded, leaving 55,397 participants. Among these, 4435 participants had MetS with exactly three components. The distributions of MetS patterns differed significantly across the FRS and ASCVD risk categories (*p* < 0.001). FWB was the most prevalent MetS pattern, followed by TFB, TFW, and TFH. In the high-risk categories, FWB remained the most prevalent MetS pattern, while TFB was also consistently represented across both FRS and ASCVD models.

### 3.2. Identifying the Predictors of CV Risk Change

As shown in Table 3, after adjustment for relevant covariates, the TFB pattern showed a consistent association with greater predicted cardiovascular risk progression at the 5-year follow-up in both the FRS and ASCVD models (FRS: HR = 1.189; ASCVD: HR = 1.144). Other MetS patterns were not significantly associated with risk progression during this period.

At the 10-year follow-up, the TFB pattern remained consistently associated with greater progression in predicted cardiovascular risk (FRS: HR = 1.204; ASCVD: HR = 1.146), followed by the TFH pattern. Similar trends were observed in the ASCVD model, with the TFB pattern consistently showing relatively higher hazard ratios compared with other patterns. The FWB pattern was also associated with increased risk progression (ASCVD: HR = 1.125). The magnitude of these associations was modest but consistent, suggesting a potentially meaningful impact at the population level. Overall, the TFB pattern appeared to be the most consistently associated with predicted cardiovascular risk progression across models and follow-up periods. This finding may reflect the combined impact of dyslipidemia, hyperglycemia, and elevated blood pressure, which are key drivers of cardiometabolic risk.

Among four-component MetS patterns (Table 4), the TFHB and TFWB patterns demonstrated consistent associations with greater progression in predicted cardiovascular risk in both models.

## 4. Discussion

In this study, we investigated the longitudinal impact of specific combinations of metabolic syndrome (MetS) components on predicted cardiovascular risk in a Taiwanese population. Individuals with MetS showed greater increases in predicted cardiovascular risk over 5- and 10-year follow-up periods. Notably, the TFB pattern was consistently associated with greater predicted cardiovascular risk progression across both models and time points, highlighting a distinct pattern beyond the traditional focus on the number of MetS components.

According to the World Health Organization’s 2018 report, CVD remains the leading cause of death [7]. Many calculators are available to estimate the predicted risk of future CV events, each designed for different populations or clinical contexts. Comparative studies suggest that the performance of these models varies across populations, with slightly higher discrimination reported for the ASCVD Risk Estimator in some settings [15]. However, other studies have shown comparable discrimination between the two models, and their relative performance may vary across populations [16]. For instance, Wang et al. (2018) found that the ASCVD Risk Estimator offered more accurate risk predictions for the mainland Chinese population [17]. In our study, the predicted CV risk generated by both the FRS and ASCVD Risk Estimator was similar, suggesting broadly consistent patterns of association and comparable risk stratification in the Taiwanese population.

In addition, we analyzed the combinations of MetS components that met four criteria to evaluate their relationship with the predicted 10-year CV risk compared to those without MetS (Table 4). Among the four-component combinations, the TFWB and TFHB patterns were significantly associated with higher predicted CV risk estimates in both models. Moebus et al. similarly found that the TG–HDL–GL–BP combination was linked to the highest predicted 10-year CV risk of myocardial infarction [18]. Accordingly, our study indicates that these two combinations share three main components—elevated triglycerides, fasting glucose, and blood pressure—with the TFB pattern. This overlap may suggest that the TFB pattern is relevant to higher long-term predicted CV risk. However, other research has offered different insights. Other research reported that over a 12.6-year follow-up, individuals with the TFH pattern had the highest CVD risk [19]. This difference may be explained by variations in study populations and methods. Lind’s study focused on a predominantly British cohort aged 40–69 years and assessed actual cardiovascular events. In contrast, our study examined predicted cardiovascular risk over time. Similarly, Stenvinkel & Shiels found that among patients with chronic kidney disease (CKD), high blood glucose, low HDL-C, and elevated triglycerides were the strongest predictors of increased CV risk [20]. However, that study evaluated individual metabolic factors rather than specific combinations and focused exclusively on a CKD population.

We also found several studies reporting results consistent with our observations for the TFB pattern. Vera et al. showed that hyperglycemia, hypertriglyceridemia, and elevated blood pressure were each individually linked to the presence of CVD [21]. Furthermore, Cui et al. (2024) found a significant interaction between the triglyceride–glucose (TyG) index and blood pressure status in relation to CVD risk [22]. Xu et al. (2024) reported that when the TyG index is elevated alongside hypertension, the risks of both CV and all-cause mortality are markedly increased [23]. Taken together, these findings suggest that MetS combinations associated with cardiovascular risk vary across studies. However, patterns involving dyslipidemia, hyperglycemia, and elevated blood pressure consistently appear important, consistent with our findings for the TFB pattern.

The exact mechanism explaining why the TFB pattern predicts future CVD risk remains unclear; however, several plausible pathophysiological explanations have been proposed. The triglyceride–glucose (TyG) index has been suggested as a surrogate marker of insulin resistance [23]. Insulin resistance may contribute to persistent hyperglycemia, which has been associated with increased glycosylation, collagen accumulation, and myocardial fibrosis, potentially impairing cardiac function [24]. In addition, insulin resistance has been linked to oxidative stress that damages vascular endothelial cells. It may also promote the growth of vascular smooth muscle cells [25]. In the context of hypertension, oxidative stress may further impair vascular function through reduced nitric oxide (NO) availability, which may contribute to endothelial dysfunction and vascular remodeling [26]. These mechanisms are likely to interact rather than act independently, with combined effects that may amplify vascular dysfunction and cardiovascular risk.

This study used a large-scale dataset and applied two well-established risk models, the ASCVD Risk Estimator and the FRS, to estimate the predicted 10-year CV risk. Unlike traditional cross-sectional studies that capture information at a single point in time, our research included longitudinal follow-ups at 5 and 10 years to examine the medium- and long-term effects of different MetS component combinations. Notably, only one specific MetS pattern consistently showed a significant link with elevated predicted CV risk across both models and time frames. From a clinical perspective, identifying specific MetS component combinations such as the TFB pattern may help improve risk stratification and guide more targeted preventive strategies in individuals at higher cardiometabolic risk.

However, several limitations should be acknowledged. First, the outcomes in this study reflected progression in model-estimated cardiovascular risk rather than observed clinical cardiovascular events. Therefore, the findings should not be interpreted as actual cardiovascular risk. Further studies with clinical outcomes are needed to confirm these associations. Second, the FRS and ASCVD risk models were originally derived from predominantly Western populations and were not specifically developed for Taiwanese individuals. Therefore, their calibration and predictive performance in this population may differ and warrant further validation. Third, dichotomizing changes in predicted cardiovascular risk may have resulted in some loss of information. However, this approach facilitates interpretation and clinical applicability. Fourth, excluding participants with inconsistent MetS component combinations may have introduced selection bias. This may limit the generalizability of our findings to individuals with more dynamic metabolic profiles. In addition, residual confounding cannot be excluded, as unmeasured or incompletely measured variables may have influenced the observed associations.

## 5. Conclusions

Specific combinations of MetS components were associated with varying levels of predicted 10-year CV risk progression. In our study, the TFB pattern was consistently associated with greater progression in predicted 10-year CV risk, according to both the FRS and the ASCVD Risk Estimator. These findings suggest that evaluating specific MetS component patterns may provide additional information beyond the total number of components when assessing predicted CV risk and may have implications for risk stratification and targeted preventive strategies. Further studies are needed to validate these findings in other populations and to examine their association with actual cardiovascular outcomes.

## Figures and Tables

**Table 1:** Baseline characteristics of study participants.

	Framingham Risk Score (2008)		*p*	ASCVD Risk Estimator (2013)		*p*
	Non-Progression	Progression		Non-Progression	Progression	
Cardiovascular risk changes, %	−1.98 ± 4.07	2.74 ± 4.75	<0.001 ***^a^	−0.75 ± 1.43	1.56 ± 2.98	<0.001 ***^a^
Sex			<0.001 ***^b^			<0.001 ***^b^
Male	5913 (23.5)	19,239 (76.5)		4801 (19.1)	20,351 (80.9)	
Female	6120 (20.2)	24,125 (79.8)		3740 (12.4)	26,505 (87.6)	
Age			<0.001 ***^b^			<0.001 ***^b^
30–49 years	8494 (19.5)	35,004 (80.5)		6425 (14.8)	37,073 (85.2)	
50–64 years	2714 (28.1)	6929 (71.9)		1693 (17.6)	7950 (82.4)	
≥65 years	825 (36.6)	1431 (63.4)		423 (18.8)	1833 (81.3)	
BMI			<0.001 ***^b^			<0.001 ***^b^
≥18.5, <24	7445 (20.9)	28,115 (79.1)		5043 (14.2)	30,517 (85.8)	
<18.5	843 (18.1)	3809 (81.9)		512 (11.0)	4140 (89.0)	
≥24, <27	2576 (24.2)	8061 (75.8)		2054 (19.3)	8583 (80.7)	
≥27	1169 (25.7)	3376 (74.3)		932 (20.5)	3613 (79.5)	
UA, mg/dL	5.50 ± 1.46	5.35 ± 1.46	<0.001 ***^a^	5.68 ± 1.49	5.32 ± 1.45	<0.001 ***^a^
LDL, mg/dL	122.47 ± 32.00	112.18 ± 26.69	<0.001 ***^a^	127.09 ± 32.71	112.10 ± 29.50	<0.001 ***^a^

UA, uric acid; LDL, low-density lipoprotein. ^a^, independent *t*-test; ^b^, Pearson chi-squared test; *** *p* < 0.001.

**Table 2:** Baseline distribution of non-MetS participants and exact three-component MetS combinations across cardiovascular risk categories. (Participants with four or five MetS components excluded.)

	n	Adjusted n (Excluding No ≤ 2)	Framingham Risk Categories, n (%)			ASCVD Risk Categories, n (%)			
			Low (<10%)	Intermediate (10–20%)	High (>20%)	Low (<5%)	Borderline (5–<7.5%)	Intermediate (7.5–<20%)	High (≥20%)
MetS pattern									
No(≤2)	50,962 (92.0%)	-	43,255 (95.9)	5199 (79.7)	2508 (66.7)	44,038 (95.2)	2723 (82.2)	3411 (74.2)	790 (63.4)
TFW	518 (0.9%)	518 (11.7)	310 (0.7)	149 (2.3)	59 (1.6)	350 (0.8)	79 (2.4)	77 (1.7)	12 (1.0)
TFH	471 (0.9%)	471 (10.6)	282 (0.6)	123 (1.9)	66 (1.8)	295 (0.6)	84 (2.5)	84 (1.8)	8 (0.6)
TFB	876 (1.6%)	876 (19.8)	288 (0.6)	288 (4.4)	300 (8.0)	376 (0.8)	124 (3.7)	296 (6.4)	80 (6.4)
TWH	106 (0.2%)	106 (2.4)	75 (0.2)	23 (0.4)	8 (0.2)	75 (0.2)	17 (0.5)	13 (0.3)	1 (0.1)
TWB	208 (0.4%)	208 (4.7)	106 (0.2)	58 (0.9)	44 (1.2)	126 (0.3)	27 (0.8)	44 (1.0)	11 (0.9)
THB	106 (0.2%)	106 (2.4)	43 (0.1)	46 (0.7)	17 (0.5)	60 (0.1)	21 (0.6)	19 (0.4)	6 (0.5)
FWH	228 (0.4%)	228 (5.1)	165 (0.4)	37 (0.6)	26 (0.7)	171 (0.4)	20 (0.6)	27 (0.6)	10 (0.8)
FWB	1516 (2.7%)	1516 (34.2)	464 (1.0)	475 (7.3)	577 (15.3)	584 (1.3)	168 (5.1)	504 (11.0)	260 (20.9)
FHB	323 (0.6%)	323 (7.3)	87 (0.2)	103 (1.6)	133 (3.5)	116 (0.3)	45 (1.4)	102 (2.2)	60 (4.8)
WHB	83 (0.1%)	83 (1.9)	42 (0.1)	19 (0.3)	22 (0.6)	49(0.1)	5 (0.2)	21 (0.5)	8 (0.6)
*p*-value			<0.001 ***			<0.001 ***			

***, *p* < 0.001. Abbreviations: T, triglycerides; F, fasting glucose; B, blood pressure; W, waist circumference; H, HDL-C. MetS patterns are indicated by the combinations they include (e.g., TFB, triglycerides + fasting glucose + blood pressure).

**Table 3:** Risk progression analysis for predicted cardiovascular risk for the three MetS patterns.

	Framingham Model				ASCVD Model			
		Adjusted HR	95% CI			Adjusted HR	95% CI	
5-Year Follow-Up			Lower	Upper			Lower	Upper
TFW vs. non-MetS	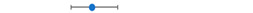	0.984	0.850	1.141	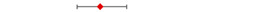	0.990	0.857	1.143
TFH vs. non-MetS	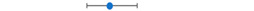	1.093	0.947	1.262	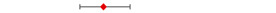	1.002	0.868	1.157
TFB vs. non-MetS	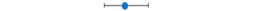	1.189 **	1.059	1.334	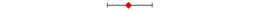	1.144 *	1.022	1.280
TWH vs. non-MetS	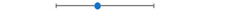	1.022	0.761	1.373	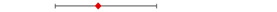	0.972	0.724	1.304
TWB vs. non-MetS	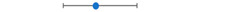	1.011	0.806	1.267	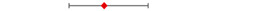	1.000	0.798	1.253
THB vs. non-MetS	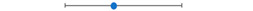	1.123	0.815	1.545	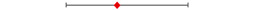	1.082	0.786	1.488
FWH vs. non-MetS	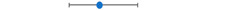	1.033	0.842	1.269	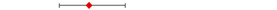	0.914	0.743	1.124
FWB vs. non-MetS	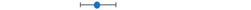	1.022	0.915	1.140	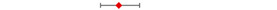	1.087	0.980	1.205
FHB vs. non-MetS	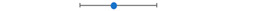	1.118	0.902	1.385	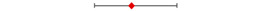	1.164	0.950	1.425
WHB vs. non-MetS	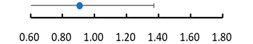	0.907	0.601	1.370	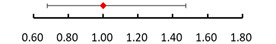	1.001	0.680	1.475
**10-year follow-up**								
TFW vs. non-MetS	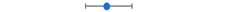	1.090	0.963	1.235	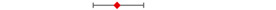	1.088	0.963	1.230
TFH vs. non-MetS	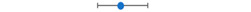	1.177 *	1.040	1.333	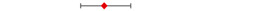	1.078	0.952	1.221
TFB vs. non-MetS	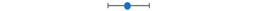	1.204 ***	1.091	1.330	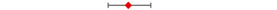	1.146 **	1.040	1.264
TWH vs. non-MetS	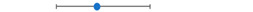	1.028	0.791	1.337	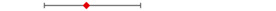	0.929	0.710	1.216
TWB vs. non-MetS	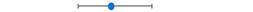	1.111	0.916	1.347	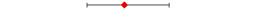	1.126	0.931	1.363
THB vs. non-MetS	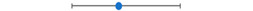	1.157	0.885	1.513	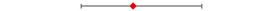	1.175	0.899	1.536
FWH vs. non-MetS	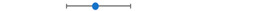	1.021	0.852	1.223	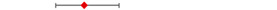	0.915	0.763	1.098
FWB vs. non-MetS	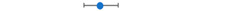	1.054	0.958	1.159	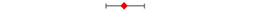	1.125 *	1.028	1.232
FHB vs. non-MetS	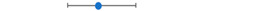	1.037	0.858	1.255	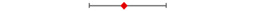	1.129	0.943	1.351
WHB vs. non-MetS	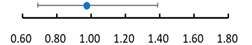	0.978	0.689	1.387	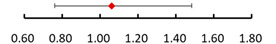	1.063	0.761	1.484

The Framingham and ASCVD models were adjusted for sex, BMI, age, uric acid, LDL-C, baseline cardiovascular risk status, and relevant clinical covariates. *, *p* < 0.05; **, *p* < 0.01; ***, *p* < 0.001.

**Table 4:** Adjusted HRs for Cardiovascular Risk by 4 MetS Pattern Combinations.

	Framingham Model			ASCVD Model		
	Adjusted HR	95% CI		Adjusted HR	95% CI	
		Lower	Upper		Lower	Upper
TFWH vs. non-MetS	1.085	0.925	1.272	0.964	0.822	1.130
TFWB vs. non-MetS	1.242 ***	1.127	1.369	1.137 **	1.033	1.251
TFHB vs. non-MetS	1.361 ***	1.157	1.600	1.300 **	1.112	1.519
TWHB vs. non-MetS	1.382 *	1.025	1.863	1.208	0.890	1.640
FWHB vs. non-MetS	1.201 *	1.005	1.435	1.184	0.999	1.403

The Framingham and ASCVD models were adjusted for sex, BMI, age, uric acid, LDL-C, baseline cardiovascular risk status, and relevant clinical covariates. *: *p* < 0.05, **: *p* < 0.01, ***: *p* < 0.001.

**Figure A1:**
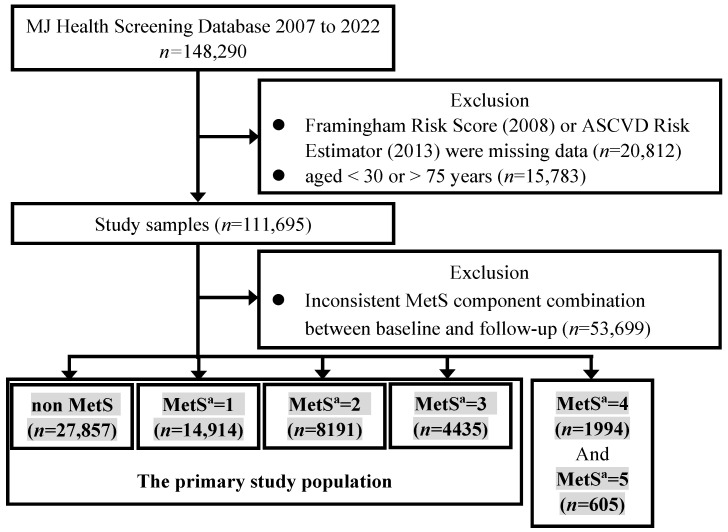
Flowchart of participant selection and inclusion criteria. a: number of abnormal MetS components; MetS was defined as the presence of three or more abnormal components.

## Data Availability

The data that support the findings of this study are available from the MJ Health Research Foundation. Restrictions apply to the availability of these data, which were used under license for the current study and are not publicly available. Data are available from the authors upon reasonable request and with permission of the MJ Health Research Foundation.
